# Full-duplex light communication with a monolithic multicomponent system

**DOI:** 10.1038/s41377-018-0083-0

**Published:** 2018-10-31

**Authors:** Yongjin Wang, Xin Wang, Bingcheng Zhu, Zheng Shi, Jialei Yuan, Xumin Gao, Yuhuai Liu, Xiaojuan Sun, Dabing Li, Hiroshi Amano

**Affiliations:** 1 Peter Grünberg Research Center, Nanjing University of Posts and Telecommunications, Nanjing, 210003 China; 2 Laboratory of Broadband Wireless Communication and Sensor Network Technology (Nanjing University of Posts and Telecommunications), Ministry of Education, Nanjing, 210003 China; 3 Department of Electronics Engineering, Zhengzhou University, Science Road 100, Zhengzhou, 450001 China; 4 State Key Laboratory of Luminescence and Applications, Changchun Institute of Optics, Fine Mechanics and Physics, Chinese Academy of Sciences, Changchun, 130033 China; 5 Institute of Materials and Systems for Sustainability, Nagoya University, Nagoya, 464-8062 Japan

## Abstract

A monolithic multicomponent system is proposed and implemented on a III-nitride-on-silicon platform, whereby two multiple-quantum-well diodes (MQW-diodes) are interconnected by a suspended waveguide. Both MQW-diodes have an identical low-In-content InGaN/Al_0.10_Ga_0.90_N MQW structure and are produced by the same fabrication process flow. When appropriately biased, both MQW-diodes operate under a simultaneous emission-detection mode and function as a transmitter and a receiver at the same time, forming an in-plane full-duplex light communication system. Real-time full-duplex audio communication is experimentally demonstrated using the monolithic multicomponent system in combination with an external circuit.

## Light communication: Quantum wells pump up the audio

A chip-scale device that uses confined light to emit and detect audio signals simultaneously has been developed by researchers from China and Japan. Recent findings have shown that quantum wells, thin semiconductors films that trap electric charges, can act as light-emitting diodes (LEDs) at the same time. It is termed the simultaneous emission-detection phenomenon. Yongjin Wang at the Nanjing University of Posts and Telecommunications and colleagues now report a technique to use light-emitting quantum wells for high-speed signal communication. The team fabricated two quantum wells on a III-nitride chip and then connected them through a suspended optical waveguide. Photocurrent measurements revealed that light generated on one side of the device could travel through the waveguide and modulate the output of the second LED. Connection to an external audio circuit enabled the quantum wells to receive sound signals and amplify them almost instantaneously.

## Introduction

It is possible to generate light from GaN-based multiple-quantum-well (MQW) diodes by injecting electrons into the MQW region, creating light with a wavelength corresponding to the smaller-bandgap material^1–4^. When approximately biased, the MQW-diode emits a broad spectrum of light. As a dual-functioning device^5,6^, the MQW-diode also functions as a photodiode that absorbs photons to liberate electron-hole pairs. Only with high-energy photons can the MQW-diode generate electron-hole pairs, which is analogous to the photoelectric effect^7^. A certain minimum frequency of the incident radiation is required for a given metal to emit photoelectrons. Therefore, there is an intriguing effect whereby the short-wavelength light emitted by the MQW-diode is absorbed by the MQW-diode itself to generate a photocurrent. This simultaneous emission-detection phenomenon is due to the spectral overlap between the electroluminescence (EL) spectra and the photocurrent responsivity spectra of the MQW-diode^8^. The self-generated photocurrent opposes a change in the injection current that produced it, indicating that self-absorption may be associated with the efficiency droop phenomenon of light-emitting diodes under high-injection conditions.

On the basis of the simultaneous emission-detection phenomenon, two MQW-diodes can be interconnected via a suspended waveguide to form a monolithic component system with integrated functionalities. Sharing an identical MQW structure and the same fabrication process flow, one MQW-diode functions as a transmitter to emit modulated light, and the other MQW-diode is used as a receiver to absorb light. Using a single MQW-diode for both the transmitter and the receiver, the light communication system is simple, compact, and flexible, particularly for on-chip data transmission. A variety of on-chip III-nitride photonic circuits have been demonstrated with various functionalities^9–13^. In particular, both MQW-diodes can serve as the transmitter and the receiver simultaneously, and a full-duplex light communication system can be established with an enhanced spectral efficiency^9^. Both MQW-diodes can communicate with each other at the same time, providing great potential for parallel sensing and transmitting of the data.

Since ultraviolet (UV) light has a higher energy than visible light, monolithic multicomponent systems show great potential for diverse applications, such as UV sensing, curing, sterilization, and on-chip power monitoring. Here we propose fabricating and characterizing a monolithic multicomponent system on a III-nitride-on-silicon platform. Both MQW-diodes are produced with an identical InGaN/Al_0.10_Ga_0.90_N MQW structure and connected to each other via a suspended waveguide. Using well-developed silicon removal and back-side III-nitride thinning techniques^14,15^, a highly confined waveguide is readily fabricated with a suspended architecture. Both MQW-diodes can operate under a simultaneous emission-detection mode at the same time, and thus real-time full-duplex audio communication using light is experimentally demonstrated by combining the monolithic multicomponent system with an external circuit.

## Results

**Fig. 1:**
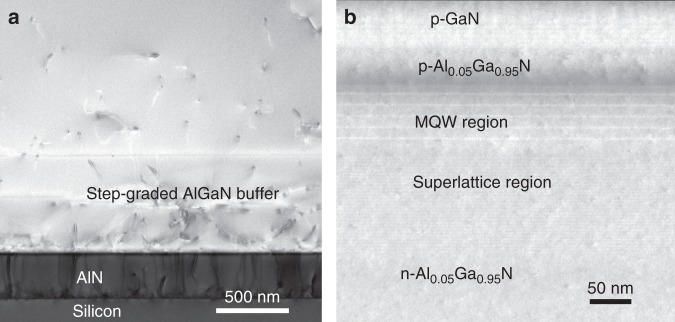
Material characterization of III-nitride epitaxial films. **a** Cross-sectional TEM image of the AlN/AlGaN multiple buffer layers. **b** High-resolution TEM image of the InGaN/Al_0.10_Ga_0.90_N MQW active region

To avoid the internal light absorption by GaN, a 2.5-μm-thick *n*-type Al_0.05_Ga_0.95_N layer is grown with Si doping of 6 × 10^18^ cm^−3^. The AlN/AlGaN buffer layers with a step-graded Al composition are first grown on a Si (111) substrate to manage the large mismatch between the lattice constants and the thermal expansion coefficients of silicon and AlGaN^16,17^. Figure 1a shows a cross-sectional transmission electron microscopy image of the AlN/AlGaN multiple buffer layers. After the growth of the thick *n*-type Al_0.05_Ga_0.95_N layer, 30 pairs of In_0.02_Ga_0.98_N/Al_0.10_Ga_0.90_N superlattice layers and 5 pairs of low-In-content InGaN/Al_0.10_Ga_0.90_N MQW structures are grown, as shown in Fig. 1b. The wavelength of the emitted light is determined by the 3-nm-thick low-In-content InGaN MQW layer, and thickness fluctuations in the InGaN layers will result in broad emission spectra. The InGaN/Al_0.10_Ga_0.90_N MQW-diode is finally formed by subsequently growing an 80-nm-thick *p*-type Al_0.05_Ga_0.95_N layer and a 10-nm-thick Mg-doped GaN contact layer.

**Fig. 2:**
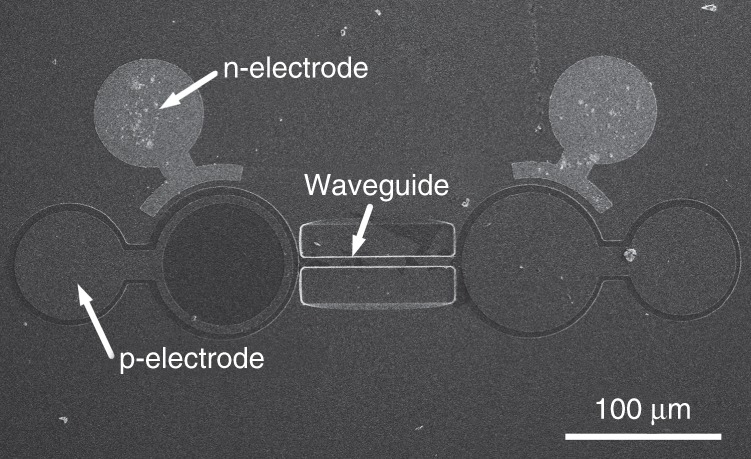
SEM image of the monolithic multicomponent system

Figure 2 shows a scanning electron microscope image of the monolithic multicomponent system. To be easily identified, one MQW-diode has a ring electrode configuration and the other has a circular electrode with a diameter of 120 µm. Both MQW-diodes having an identical MQW structure are monolithically integrated together through the 8-µm-wide and 130-µm-long suspended waveguide. The suspended waveguide is essential for the in-plane light coupling between the waveguide and the MQW-diodes^13,18^. Because of the large index differences between air and the GaN in the system, the light can be highly confined inside the suspended waveguide by total internal reflection. Hence, the light emitted from one MQW-diode is coupled into the suspended waveguide. Then, the guided light travels along the suspended waveguide and is finally sensed by the other MQW-diode. The light generated in the MQW-diode radiates almost uniformly in all directions, which affects the light coupling between the waveguide and the MQW-diodes in the monolithic multicomponent system. Recently, Sun et al. demonstrated a continuous-wave electrically injected III-nitride laser diode directly grown on a silicon substrate^19–22^. Since the III-nitride laser diode on silicon has a waveguiding structure by growing a thick low-index cladding layer, the monolithic III-nitride multicomponent system can be produced without silicon removal, and the coupling efficiency between the waveguide and the MQW-diode will be improved.

**Fig. 3:**
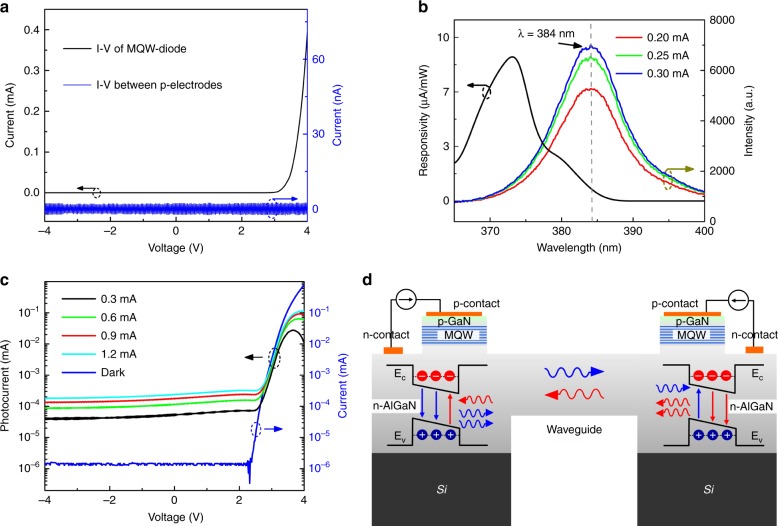
Optical and electrical performance of the monolithic multicomponent system. **a** Measured *I*–*V* curves of the monolithic multicomponent system. **b** EL spectra and spectral responsivity of the ring MQW-diode. **c** Induced photocurrent at the circular MQW-diode as a function of the injection current of the ring MQW-diode. **d** Schematic of full-duplex light communication of the monolithic multicomponent system using identical MQW-diodes

Figure 3a shows current–voltage (*I–V*) plots of the monolithic system, in which the ring MQW-diode exhibits a turn-on voltage of 3.0 V. The high resistance between the two *p*-electrodes confirms that both MQW-diodes can be used as fully independent devices. The EL spectra of the ring MQW-diode are shown in Fig. 3b, exhibiting a dominant EL peak at 384 nm. After silicon removal, the EL spectra of a suspended MQW-diode shift due to the change in built-in stress^23,24^, providing a feasible way to increase the spectral overlap between the emission and detection spectra^25^. An increase in injection current from 0.2 to 0.3 mA increases the intensity of the emitted light. Using a micro-transmittance setup, the responsivity spectra are characterized. There is a 40-nm-wide spectral overlap region, suggesting that the emitted light at short wavelengths can be absorbed by the MQW-diode itself to liberate electron-hole pairs. The conversion efficiency is limited because only high-energy light photons can be absorbed by the other MQW-diode. Since the intensity of the emitted light is proportional to the injection current of the MQW-diode, an increase in intensity of the emitted light increases the magnitude of the self-generated photocurrent when the injection current is increased for the MQW-diode. Because both MQW-diodes are interconnected by the suspended waveguide in the monolithic multicomponent system, the circular MQW-diode can absorb high-energy photons emitted from the ring MQW-diode to generate a photocurrent. According to the EL spectra shown in Fig. 3b, the intensity of the emitted light is proportional to the injection current of the ring MQW-diode. As a result, a change in injection current of the ring MQW-diode can modulate the photocurrent of the circular MQW-diode. Figure 3c shows the normalized photocurrent of the circular MQW-diode. The photocurrent changes with the injection current of the ring MQW-diode, endowing the monolithic multicomponent system with the functionality of in-plane light communication. Figure 3d schematically illustrates the full-duplex light communication mechanism of the monolithic multicomponent system using identical MQW-diodes. When two identical MQW-diodes are produced on a single chip, one MQW-diode functions as a transmitter to emit light, and the other MQW-diode serves as a receiver to absorb light. When identical MQW-diodes emit light at the same time, they absorb high-energy light photons to produce electron-hole pairs, leading to a change in internal electric voltage across the junction. The simultaneous emission-detection phenomenon occurs, and full-duplex data transmission is established in the monolithic multicomponent system.

## Discussion

**Fig. 4:**
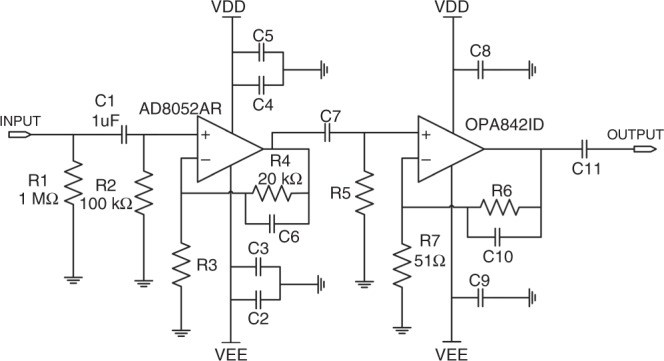
External filter and amplifier circuit

The received signals can be directly recovered when the circular MQW-diode operates as a photodiode. However, the circular MQW-diode will mix the received signals with the driven electrical signals under simultaneous emission-detection conditions. An external circuit is employed to filter and amplify tiny electrical signals, so they can be measured and characterized by a digital storage oscilloscope. As shown in Fig. 4, the original current signals are converted to electrical voltage signals after the resistance *R*_1_ of 1 MΩ. Owing to the blocking capacitance *C*_1_ and resistance *R*_2_, the direct electrical signals are filtered out. An AD8052AR amplifier is used to amplify the electrical voltage signals by approximately 10-fold, where *R*_2_ and *R*_3_ are 100 and 1 kΩ, respectively. An OPA8421D amplifier is used to subsequently amplify the output signals, and a sliding rheostat *R*_6_ is used. The whole amplifier circuit can amplify the signals by a factor up to nearly 600. Finally, an Agilent DSO9254A digital storage oscilloscope is used to characterize the output signals.

**Fig. 5:**
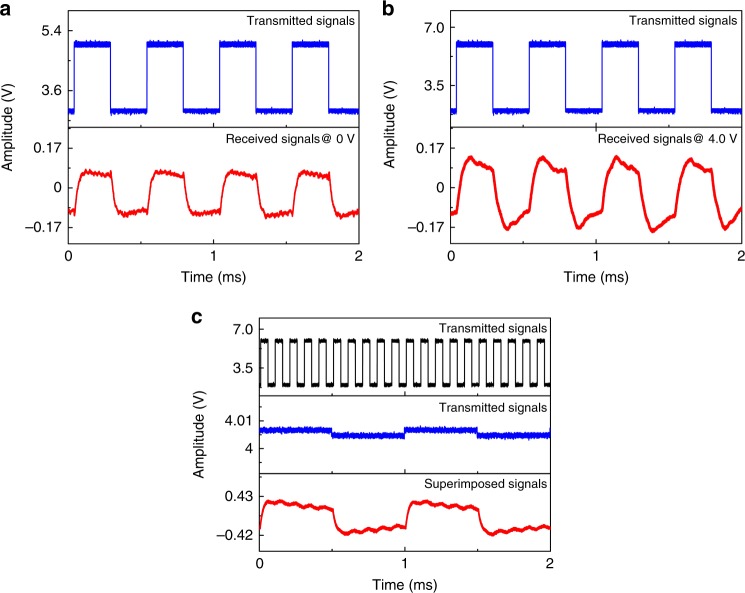
Different operation conditions of the monolithic multicomponent system. **a** Square-wave signals received at the circular MQW-diode with zero bias. **b** Square-wave signals received at the circular MQW-diode with a bias voltage of 4.0 V. **c** Superimposed signals under the simultaneous emission-detection condition

An arbitrary waveform generator directly drives the ring MQW-diode functioning as the transmitter, causing it to emit the modulated light, and the circular MQW-diode serves as the receiver to sense the guided light inside the suspended waveguide. Thus, in-plane light communication is established. For a given III-nitride-on-silicon wafer, the bandwidth of the MQW-diode can be improved by reducing the resistance-capacitance time. Without modifying the MQW-diode structure, the frequency response is dependent on the voltage applied. A variety of monolithic multicomponent systems have demonstrated hundreds of Mbps data transmission rate using non-return-to-zero on-off-keying modulation. The transmitter is modulated with a peak-to-peak voltage *V*_pp_ of 1.0 V and an offset voltage *V*_offset_ of 2.0 V, and square-wave signals are loaded. When the receiver operates under zero bias, it only functions as a photodiode. Fig. 5a shows the received signals at a frequency of 2 kHz, which are characterized using a DC coupling model with an input impedance of 1 MΩ. When the receiver is turned on with a stable bias voltage of 4.0 V, it emits light and simultaneously senses the modulated light from the transmitter. The measured signals merge the received signals and the directly driven electrical signals. The external circuit filters out the directly driven electrical signals and amplifies the received signals. Fig. 5b illustrates the received signals after the external circuit. When the receiver is modulated at a frequency of 1 kHz to emit light, full-duplex light communication is established. The superimposition of the received and transmitted signals occurs. Fig. 5c illustrates the superimposed signals after the external circuit. The received signals can be extracted through a self-interference cancellation method, bringing full-duplex communication closer to reality^26^.

**Fig. 6:**
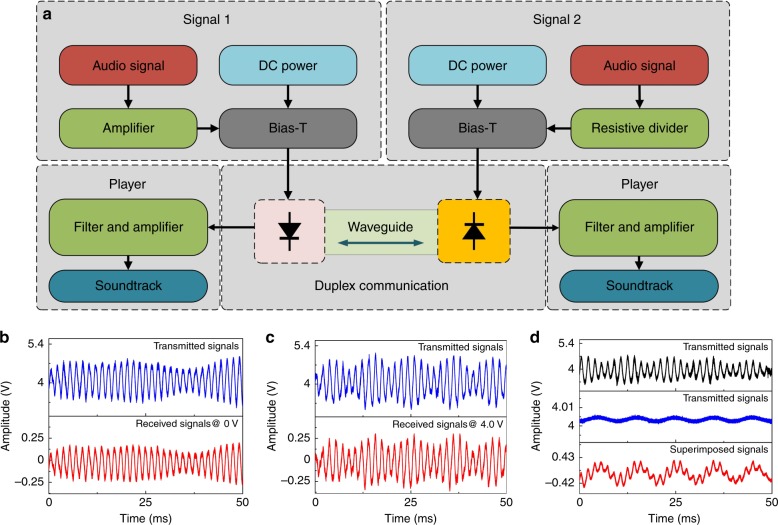
Full-duplex audio communication using the monolithic multicomponent system. **a** Schematic of full-duplex audio communication using light. **b** Audio signals received at the circular MQW-diode with zero bias. **c** Audio signals received at the circular MQW-diode with a bias voltage of 4.0 V. **d** Superimposed signals under the simultaneous emission-detection condition

Figure 6a schematically illustrates real-time full-duplex audio communication by combining the monolithic multicomponent system with an external circuit. The audio signals are employed to modulate the MQW-diodes through bias-tee circuits. Fig. 6b shows the amplified audio signals after filtering when the circular MQW-diode is biased at 0 V. The output signals are sent to an audio player. When biased at 4.0 V, the circular MQW-diode emits light and receives audio signals simultaneously. The amplified signals are demonstrated in Fig. 6c. When the circular MQW-diode is also modulated by audio signals, real-time full-duplex audio communication is established because both MQW-diodes can transmit and receive audio signals at the same time. Fig. 6d illustrates the superimposition of the received and transmitted audio signals. The supplementary video, for the first time, experimentally demonstrates full-duplex audio communication using light, in which the music broadcast by the audio player is mixed.

In conclusion, the simultaneous emission-detection behavior observed in the proposed monolithic multicomponent system is due to the overlap of the EL spectra and the responsivity spectra of the MQW-diodes. The monolithic multicomponent system is composed of two InGaN/Al_0.10_Ga_0.90_N MQW-diodes interconnected by a suspended waveguide, and both MQW-diodes can simultaneously communicate with each other using light, leading to the formation of an in-plane light communication system. Real-time full-duplex audio communication is experimentally demonstrated, providing a feasible approach to developing monolithic multicomponent systems with integrated functionalities for diverse applications.

## Materials and methods

The monolithic multicomponent system is implemented using a four-mask process: (1) in the first step, MQW-diode mesas are patterned and etched down to the *n*-type Al_0.05_Ga_0.95_N; (2) following electron beam evaporation, a lift-off process is conducted to form 20/200 nm Ni/Ag *p*- and *n*-electrodes; (3) a waveguide for optically interconnected MQW-diodes is formed; and (4) in the final step, back-side processes are performed to remove silicon substrate beneath the multicomponent system and then to thin suspended III-nitride slab without etch hard mask, resulting in a completely suspended waveguide.

## Electronic supplementary material

full-duplex audio communication
